# Dynamics and genetics of a disease-driven species decline to near extinction: lessons for conservation

**DOI:** 10.1038/srep30772

**Published:** 2016-08-03

**Authors:** M. A. Hudson, R. P. Young, J. D’Urban Jackson, P. Orozco-terWengel, L. Martin, A. James, M. Sulton, G. Garcia, R. A. Griffiths, R. Thomas, C. Magin, M. W. Bruford, A. A. Cunningham

**Affiliations:** 1 Institute of Zoology, Zoological Society of London, Regent’s Park, London NW1 4RY, UK; 2 Durrell Institute of Conservation and Ecology, School of Anthropology and Conservation, University of Kent, Canterbury, Kent CT2 7NR, UK; 3 Durrell Wildlife Conservation Trust, Les Augres Manor, Trinity, Jersey, Channel Islands, UK; 4 Department of Life Sciences, Imperial College London, Silwood Park Campus, Buckhurst Road, Ascot, Berkshire SL5 7PY, UK; 5 School of Biosciences, Cardiff University, The Sir Martin Evans Building, Museum Avenue, Cardiff CF10 3AX, UK; 6 Department of Biology & Biochemistry, University of Bath, Claverton Down, Bath BA2 7AY, UK; 7 Department of Environment, Montserrat Ministry of Agriculture, Housing, Lands and Environment, Montserrat, West Indies; 8 Forestry, Wildlife and Parks Division, Dominica Ministry of Agriculture and Fisheries, Botanical Gardens, Roseau, Commonwealth of Dominica, West Indies; 9 Chester Zoo, Cedar House, Caughall Road, Upton by Chester, Chester CH2 1LH, UK; 10 Division of Agriculture, Dominica Ministry of Agriculture and Fisheries, Botanical Gardens, Roseau, Commonwealth of Dominica, West Indies; 11 The Royal Society for the Protection of Birds, The Lodge, Sandy, Bedfordshire SG19 2DL, UK; 12 Sustainable Places Research Institute, Cardiff University, Cardiff CF10 3BB, UK

## Abstract

Amphibian chytridiomycosis has caused precipitous declines in hundreds of species worldwide. By tracking mountain chicken (*Leptodactylus fallax*) populations before, during and after the emergence of chytridiomycosis, we quantified the real-time species level impacts of this disease. We report a range-wide species decline amongst the fastest ever recorded, with a loss of over 85% of the population in fewer than 18 months on Dominica and near extinction on Montserrat. Genetic diversity declined in the wild, but emergency measures to establish a captive assurance population captured a representative sample of genetic diversity from Montserrat. If the Convention on Biological Diversity’s targets are to be met, it is important to evaluate the reasons why they appear consistently unattainable. The emergence of chytridiomycosis in the mountain chicken was predictable, but the decline could not be prevented. There is an urgent need to build mitigation capacity where amphibians are at risk from chytridiomycosis.

Recent studies indicate that the Earth has entered a sixth period of mass extinction^1^. Unlike previous mass extinction events, the current situation stems from human activities and mitigation measures have been agreed upon internationally in the form of the Convention on Biological Diversity (CBD). This treaty’s undertakings include the prevention of extinction of known threatened species and the safeguarding of genetic diversity for a range of organisms, including those of socio-economic or cultural value. The Aichi 2020 Targets for the CBD specify a range of measures, including those designed to “improve the status of biodiversity by safeguarding ecosystems, species and genetic diversity” (www.cbd.int). These targets were driven by a generally perceived failure of the international community to achieve the CBD’s goal of halting biodiversity loss by 2010, yet it is becoming apparent that many of the 2020 targets will also be missed^2^. It is important to evaluate the reasons why these targets appear consistently unattainable if lessons are to be learned. Within this context, case studies of success and failure in conservation planning and action can provide important examples of the challenges of meeting global biodiversity targets and on how success rates may be improved.

With over 40% of species currently threatened with extinction^3^,^4^, amphibians are a disproportionately affected group. This rate of loss is increasing^5^, with emerging infectious diseases, and specifically amphibian chytridiomycosis, being one of the primary drivers of this unprecedented level of threat^5^,^6^. Amphibian chytridiomycosis, due to infection with the non-hyphal chytrid fungus, *Batrachochytrium dendrobatidis* (Bd), is thought to have caused the decline or extinction of over 200 species of amphibian world-wide in recent decades^6^,^7^.

Chytridiomycosis-induced amphibian declines usually occur following the introduction of Bd to a naïve population. The precise timing of disease introduction, however, is rarely identified and often the first recognition of Bd emergence in an amphibian population is the loss or rapid decline of that species at a study location^8^,^9^. As a result, few studies have captured the trajectory and rate of chytridiomycosis-driven amphibian declines from the time of disease onset. Where data are available, population collapses of more than 90% in as little as 1–3 years from first recorded death have been reported repeatedly^8^,^9^,^10^,^11^,^12^,^13^,^14^.

A lack of monitoring and the speed of chytridiomycosis-driven amphibian declines have resulted in few data being available for assessing the genetic impact of the disease. Despite the numerous population declines reported, only one study has investigated the associated genetic impact, providing evidence of a disease-induced population bottleneck in the common midwife toad (*Alytes obstetricans*)^15^. This is important as amphibians, which often have small effective population sizes, fragmented populations and low dispersal rates, are particularly vulnerable to loss of genetic diversity^16^. The importance of conserving genetic diversity has recently been recognised by its inclusion in the CBD’s 2020 Targets (Strategic Goal C, Target 13).

The mountain chicken (*Leptodactylus fallax*) is the largest frog endemic to the Lesser Antilles which can live 12 years^17^. This species is now confined to only two islands, Montserrat and Dominica, after being driven to extinction on other east Caribbean islands by introduced predators and over-hunting for food prior to the global emergence of Bd^18^,^19^,^20^,^21^. With relevance to the CBD’s 2020 Target 13, the species is culturally valuable; it features on Dominica’s official coat-of-arms and is incorporated into local folklore and proverbs. The mountain chicken was also of socio-economic importance as, prior to the current declines, the frog was the national dish of Dominica and was a source of income for hunters, restaurants and the tourism industry. A monitoring programme for the mountain chicken was initiated in 1998 on Montserrat and in 2002 on Dominica by Fauna & Flora International and the respective Forestry Departments to investigate the impacts of hunting that, along with invasive rats and pigs, was considered the main threat to the species^19^.

In December 2002, reports of dead and sick mountain chickens were first received by forestry officers from members of the public in Dominica. Initially, only isolated reports were made but within weeks widespread mortality was apparent. A diagnosis of chytridiomycosis due to Bd infection was made on carcases shipped to the Zoological Society of London for pathological examination^22^. Following the epidemic on Dominica, the mountain chicken was listed as critically endangered by the IUCN in 2004^23^. Targeted surveillance of amphibians in 2005 showed Bd to be absent from Montserrat^24^, therefore a risk analysis was conducted to identify potential pathways for introduction of the pathogen to the island. The highest risk identified was the accidental importation of infected tree frogs (*Eleutherodactylus* spp.) within shipments of produce, most of which was imported directly from Dominica^25^. Recommendations were made and communicated to minimise this risk, including the removal of any amphibians found in produce prior to shipping, the capture and euthanasia (in contrast to the common practice of release into the wild) of any amphibians found in produce on arrival in Montserrat, and awareness-raising for exporters, importers and the public^25^. However, in February 2009, Montserrat forestry officers reported multiple dead mountain chickens during a routine visit to one of the population monitoring sites. We rapidly diagnosed the cause as chytridiomycosis due to infection with Bd by histopathology which has been more recently verified by real-time PCR on archived samples (authors’ unpublished data). Further investigations showed evidence of an eastward wave of mass mortality across the species’ range on Montserrat. In response, 50 mountain chickens were captured from the last intact population in front of the epidemic wave to set up a biosecure conservation assurance population in Europe.

On both Dominica and Montserrat, long-term monitoring programmes produced mountain chicken population demographic data and genetic samples before, during and after the onset of epidemic chytridiomycosis. Here, we quantify the population and genetic impacts of the disease, discuss the management responses to this crisis and evaluate their effectiveness in terms of conserving biodiversity. This study of the predictable near-extinction of a culturally iconic species due to a process addressed by Aichi Target 9 (invasive species and pathways) is a useful model for understanding how failures in national and international governance and support mechanisms are impeding our ability to meet the CBD 2020 targets.

## Results

### Demography and disease

Detailed pathological examinations were conducted on four adult frogs found dead between 19^th^ February and 28^th^ March 2003 from three sites across Dominica. Histological examination of hind-leg skin and feet showed large numbers of intracellular sub-spherical, septate structures characteristic of Bd^26^. All of the extracted DNA samples tested positive for Bd DNA using qPCR (4567, 4780 and 12643 genomic equivalents). No other findings that could be related to the cause of death were observed. Twenty-eight additional mountain chickens were found dead and 21 found alive with signs of severe chytridiomycosis (See Supplementary Table 1 for more details).

Dead mountain chickens were first reported on Montserrat in February 2009 on the eastern side of the island. Twelve transects were surveyed across the island during the following two weeks, and epidemic mortality was observed at three sites in the east. No mountain chickens were found at five long-term population monitoring sites in the north or west of the Centre Hills, and severely depleted populations at sites in the south and east. Only two sites, Fairy Walk and Corbett Spring, were found to contain apparently healthy mountain chicken populations. Both of these sites are in the extreme south-east of the Centre Hills. Using qPCR, we confirmed the presence of Bd DNA in skin swabs at five of the seven sites in which mountain chickens were found, including all five with observed mortality. Only skin swabs from Corbett Spring and Fairy Walk did not test positive for Bd DNA. On returning to Montserrat in August 2009, no mountain chickens were found at any site other than Fairy Walk and one other site nearby, where epidemic mortality was observed. Of 120 mountain chickens skin-swabbed during two weeks of monitoring at Fairy Walk in August 2009, 105 (87.5%) tested positive for Bd DNA. The temporo-spatial pattern of mortality and decline suggested a north-west to south-east epidemic wave with an active front in the remaining populations in the south-east.

**Figure 1:**
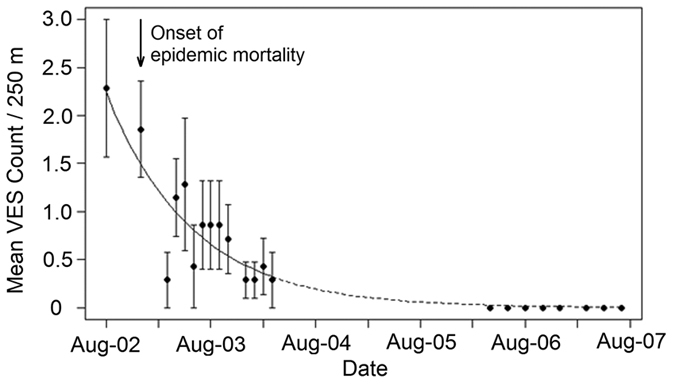
Generalised linear mixed effects model of encounter rate of mountain chickens on Dominica. Dashed line indicates model extension to cover period in which no data were collected and the data not included in the model. Error bars represent standard error around the mean of count across the transects.

No explanatory variables other than time were retained in the final generalised linear mixed effects model (GLMM) of the Dominica decline (Chi-sq = 20.31, df = 1, p = 0.01). Mean visual encounter rates per 250 m declined by more than 50% from 2 (SE = 0.31) to 0.85 (SE = 0.18) within 12 months from August 2002 (Fig. 1). By March 2004, encounter rates on the transects had fallen to 0.29 (SE = 0.11), a decline of c. 85% in 18 months. When the surveys restarted in 2006 no frogs were seen on any transect, despite increased efforts, until 2014 when a single population of 14 individuals was found on one of the original transects.

**Figure 2:**
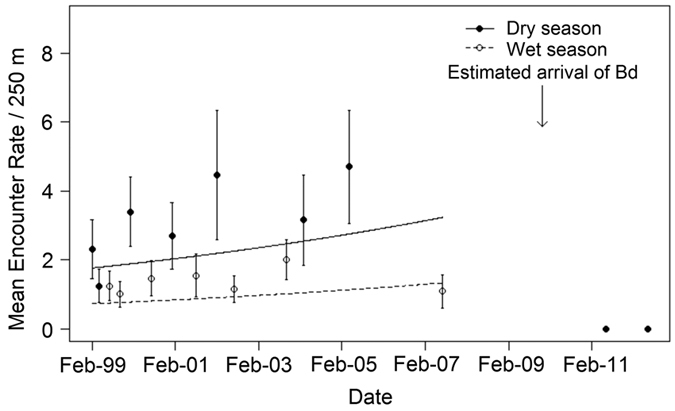
Generalised linear mixed effects model of mountain chicken encounter rate on historically monitored transects on Montserrat. Error bars represent the SE around the mean encounter rate across the transects.

On Montserrat, season (Likelihood ratio test (LRT) Chi-sq = 20.997, df = 1, p < 0.0001) and time (LRT Chi-sq = 3.8476, df = 1, p = 0.04) were retained as fixed effects in the GLMM for the 1999–2007 pre-decline data. A greater number of individuals were encountered on transects during the dry than the wet season, and the visual encounter rate increased between 1999 and 2007 (Fig. 2). However, visual encounter surveys (VES) in June 2011 and 2012 did not detect any mountain chickens.

**Figure 3:**
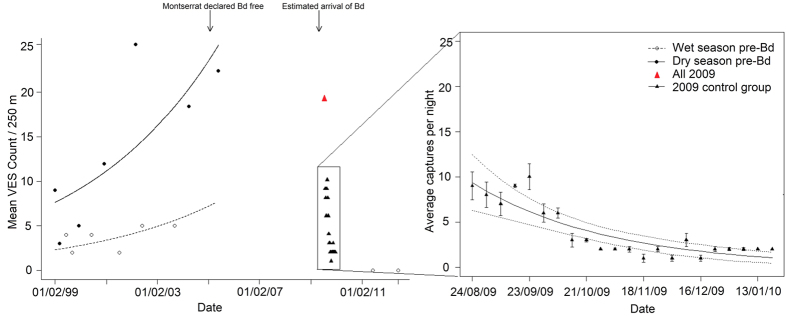
Decline in mountain chicken encounter rate at Fairy Walk on Montserrat. LHS shows general linear model of historical monitoring and RHS shows close up of general linear model of rapid decline during epidemic chytridiomycosis observed in 2009. Red triangle shows total encounter rate in first week of intensive monitoring during the study described in ref. 42. Black triangles indicate only the control groups (~50% of the population) to exclude the impact of the treatment used in ref. 42.

Time was the only significant parameter retained in the generalised linear model (GLM) of the Fairy Walk decline (z = −5.41, df = 21, p < 0.0001), with the encounter rate per 250 m of transect declining from a maximum of 10 (week 5) individuals to 2 (week 18) a decrease of c. 80% in just 13 weeks (Fig. 3). Continued monitoring of the Montserrat transects, 2012–2014, identified only four individuals at three sites.

### Range change estimates

**Figure 4:**
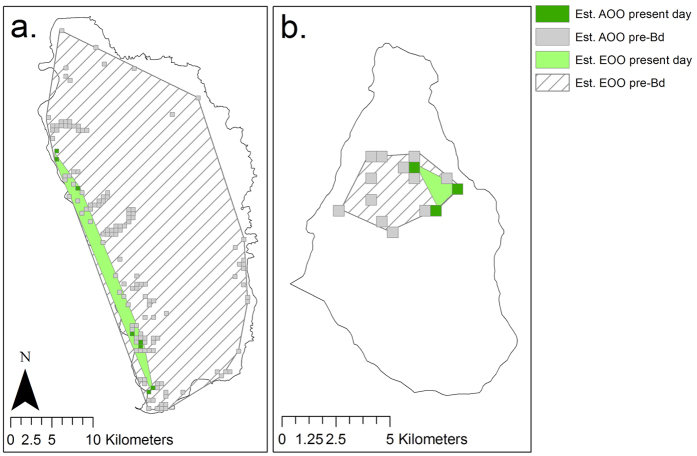
Mountain chicken range collapse on (**a**) Dominica and (b) Montserrat. Area of occupancy is shown as grid squares and extent of occurrence as a minimum convex polygon. Figure created in Esri ArcMap 10.2. under license (www.esri.com).

Intensive surveys on Dominica in 2014 encountered a total of 44 mountain chickens across six sites. On Dominica, the estimated extent of occurrence (EOO) of the mountain chicken was reduced by 95% (from 663.63 km^2^ to 38.08 km^2^) and the estimated area of occurrence (AOO) was reduced by 94.4% (from 35.25 km^2^ to 1.75 km^2^) between 2002 and 2014 (Fig. 4). The mountain chicken population caught in 2014 was Bd-negative and it is possible that a degree of nascent post-epidemic population recovery was present.

The island-wide decline on Montserrat, 2009 to 2012, reduced the species’ estimated EOO by 87.9% (from 12.68 km^2^ to 1.53 km^2^) and the estimated AOO by 80% (from 3.75 km^2^ to 0.75 km^2^; Fig. 4). There is no apparent population recovery on this island to date.

### Population genetics

Analysis of eight microsatellites showed a total of 52 alleles (Dominica: 48, Montserrat: 38) with an average of 6.5 per locus and 34 shared between both islands (Table 1). The expected heterozygosity ranged from 0.39 to 0.75 in Dominica and from 0.48 to 0.75 in Montserrat. Microchecker analysis identified the markers Lepfal_0867, Lepfal_3035, Lepfal_1628 and Lepfal_3956 as having potential null alleles in Dominica. The same analysis within Montserrat identified markers Lepfal_1628 and Lepfal_3956 as potentially having null alleles. As Lepfal_1628 and Lepfal_3956 show high probability of null alleles in both islands, we carried out analyses to assess the effect of the missing alleles on the summary statistics describing the genetic variation in these populations. The results for both analyses were similar with no statistical associations changing significance and/or direction, with the exception of the number of alleles per locus (uncorrected for sample size). When comparing the number of alleles in *pre-* and *post-* decline populations within Dominica using the remaining six loci, the difference was not significant, but with eight loci (including the null alleles) there was a significant decline in diversity (p = 0.03). We present the results including all markers for the remainder of this section. No significant linkage disequilibrium was found between any pair of loci in each island’s population.

To avoid the confounding effects of rapid population decline on genetic diversity, the interisland genetic comparison was conducted using only samples collected prior to the arrival of Bd. The comparison between Dominica and Montserrat *pre-decline* showed no significant differences in expected heterozygosity (Welch t-test p = 0.727; Table 1). However, the allelic richness (corrected for differences in sample size) within the Dominican *pre-decline* population showed a consistent but non-significant trend towards a higher allelic richness than the *pre-decline* population on Montserrat (Table 1; Welch t-test p = 0.06). The population on Montserrat was not in Hardy-Weinberg equilibrium (HWE) due to three loci showing significant excess of homozygosity and one locus showing a significant excess of heterozygosity (HWE exact test, p = 0.016, Supplementary Table 2). Consistent with these results, the inbreeding coefficient of Montserrat was significantly positive (p < 0.001), and was not significantly different than that of the Dominican datasets (Welch t-test, *pre-decline* Dominica comparison p = 0.814; *post-decline* Dominica comparison p = 0.421; Table 1). Differentiation between the *pre-decline* Dominican vs. *pre-decline* Montserrat and *post-decline* Dominican vs. *pre-decline* Montserrat datasets showed significant F_ST_ values of 0.2 and 0.25 respectively (p < 0.001).

Within Dominica, the expected heterozygosity and allelic richness (corrected for sample size) for samples collected from 2002 to 2006 (*pre-decline*, n = 29) were both greater than those of the wild individuals sampled in 2014 (*post-decline*, n = 17). Expected heterozygosity declined from 0.61 to 0.49 and allelic richness reduced from 5.2 to 3.5, although these differences were not significant (Table 1; Welch t-test p = 0.17 and p = 0.05 respectively). Hardy Weinberg exact tests indicated that neither dataset was in equilibrium, with heterozygote deficiency at four loci in the *pre-decline* dataset (p = <0.05) and three loci in the *post-decline* dataset (p = <0.05; Supplementary Table 2). We also observed significant positive inbreeding coefficients in the *pre-* and *post-* decline populations (Table 1), although they were not significantly different between the *pre-* and *post-* decline datasets (Welch t-test p = 0.493). While these results suggest a decrease in genetic diversity after the population crash, demographic analyses using Bottleneck did not find evidence for a genetic bottleneck in the *post-decline* population on Dominica (Heterozygote excess Wilcoxon’s Sum Rank test TPM p = 0.58 and SMM p = 0.88).

### Representativeness of the founder population

Genetic diversity and differentiation was assessed to ascertain whether the individuals removed from Montserrat in 2009 to establish the captive breeding program (*Founders*, n = 11) were a genetically representative sample of the pre-epidemic wild Montserrat individuals (*Wild*, n = 74). The *Founders* and *Wild* populations were both overall within HWE. The differences in average number of alleles per locus, allelic richness and heterozygosity between these populations (Table 1), were not statistically significant (all Welch t-tests: p > 0.05). The *Wild* population had a positive inbreeding coefficient (F_IS_ = 0.18) that was significantly larger than zero (p < 0.001), but which did not statistically differ from that in the *Founder* population (Welch t test p = 0.705, Table 1). As expected from these results, the differentiation between the *Wild* and *Founder* individuals resulted in an F_ST_ value of 0.

**Figure 5:**
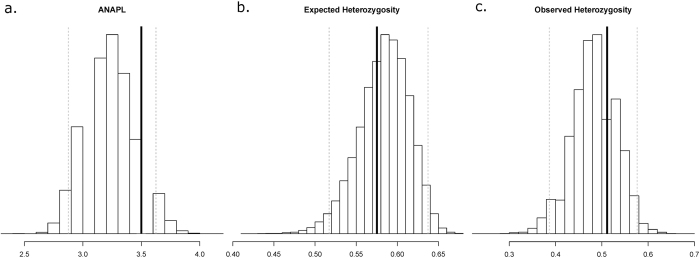
Histograms of the expected distributions of three genetic variation statistics. Each histogram describes the expected distribution of a summary statistic in 100,000 bootstrap resamples of eleven individuals in the wild population. The bold line is the observed value of the statistics in the eleven founders, and the grey dotted lines show the 2.5th and 97.5th percentiles of the distribution. (**a**) Average number of alleles per locus–ANAPL; (**b**) Expected Heterozygosity; (**c**) Observed Heterozygosity.

The genetic variability of the *Founder* population used for captive breeding was compared to the *Wild* population accounting for sample size using 100,000 random samples of eleven animals among the *Wild* samples. The average number of alleles per locus, the observed and the expected heterozygosity (Table 1) fell well within the 2.5 (Lower Boundary – LB) and 97.5 (Upper Boundary – UB) percentiles of the distribution of those statistics in the random samples of the *Wild* population. The range between the LB and UB for these statistics was 2.9–3.6 (average number of alleles per locus), 0.39–0.58 (observed heterozygosity), and 0.52–0.64 (expected heterozygosity). Thus, the resampling analysis showed that the genetic variation captured by the *Founder* animals was within the expected distribution of genetic variation in the *Wild* population (Fig. 5).

## Discussion

This study shows that the first recorded outbreak of chytridiomycosis in the Lesser Antilles caused the rapid decline and near extinction of the mountain chicken on Montserrat and Dominica. The decline of the mountain chicken across its range is amongst the fastest recorded for any species, with island-wide population collapses due to chytridiomycosis occurring within 18 months on Dominica and under one year on Montserrat. On Dominica, the visual encounter rate halved every seven months between August 2002 and March 2004. It is not clear when the mountain chicken population reached undetectable levels, but by 2006 no individuals could be found despite repeated efforts in prime habitat. A similar but more rapid trajectory of decline was observed on Montserrat. The last intact population on this island, at Fairy Walk, was monitored intensely from August 2009 shortly after chytridiomycosis reached this site. Initial encounter rates were similar to those seen pre-epidemic (between 1999 and 2007), but the encounter rate halved every seven weeks until monitoring had to be suspended after five months due to volcanic activity.

Detectability of mountain chickens on transects was likely not 100%. We attempted to fit an open population N-mixture model^27^ to account for detection probability, but these models failed a goodness of fit test and were not used in analysis despite producing similar estimates for the rate of decline as those produced by the GLMM. Despite this inability to assess detection probability, we maintain that VES is an effective tool for measuring terrestrial amphibian abundance especially when there are a large number of sites in a variety of habitats^28^,^29^,^30^. Although presence/absence surveys may have allowed an even greater number of sites to be surveyed, they do not account for unequal abundance between sites^31^, which was evident for this species from pre-decline transects. The population coverage was also maximised as transects were originally set up to monitor its status across both islands and so were positioned in known population sites that covered the geographical extremes of the species range.

Amphibian chytridiomycosis has caused similarly rapid declines in other populations^8^,^9^,^10^,^11^,^12^,^14^, but rarely have entire species declines across their entire range been recorded within such a short time frame (85% in 18 months on Dominica, 85% in 13 weeks at Fairy Walk on Montserrat). No other threat has been recorded as having driven such a rapid range-wide decline of a species. The brown tree snake on Guam caused site level extirpations of several bird species within 18 months, however range-wide declines occurred more slowly^32^. The saiga antelope (*Saiga tatarica*) declined by 95% over 15 years due to hunting^33^ and the Oriental white-rumped vulture (*Gyps bengalensis*) declined in India by 99.9% over 15 years as a result of secondary poisoning^34^.

The range collapse of the mountain chicken was also precipitous, with a decline of over 80% in both AOO and EOO on Montserrat and of ~95% on Dominica. The species’ range on Dominica has collapsed to a small part of the west coast and has undergone severe fragmentation. On Montserrat, only four individuals at three sites have been discovered since 2012 despite repeated intensive surveys; since 2014 only two of these individuals have been recaptured, both at the same site. While the persistence of individuals after a chytridiomycosis epidemic has been recorded in other studies^35^,^36^,^37^,^38^, the extent of the mountain chicken range collapses are similar to those reported in ref. 14, where the majority of mountain yellow-legged frog (*Rana muscosa*) populations were extirpated over a four year period by epidemic chytridiomycosis. The reduction in mountain chicken population connectivity, however, undoubtedly affects the level of gene flow between remaining individuals, increasing the risk of extinction^16^,^39^.

The optimal temperature for the growth of Bd is 17–25 °C^40^ and this is why montane amphibians in the tropics are thought to be at greatest risk^41^. In Dominica mountain chickens occur in the lowlands where temperatures regularly exceed 28 °C (authors’ unpublished data) at which Bd stops growing^40^. It is not clear therefore why this species has been so badly affected by chytridiomycosis on this island. One possible mechanism might be if the behaviour of this species maintains a lower temperature microclimate in burrows or other refugia. Also, when sick with chytridiomycosis, mountain chickens seek water bodies^42^, which in Dominica are primarily rivers fed by cool mountain streams.

Previous analyses of mitochondrial DNA sequence data (mtDNA; cytochrome B; 804 bp) for the mountain chicken (Fig. 3 within ref. 20), found a single haplotype across the islands (n = 2). Our own analysis of a 463 bp portion of the cytochrome oxidase 1 mtDNA gene corroborates this finding (DNA sequences provided in Supplementary data 1), strongly indicating that Dominican and Montserrat mountain chickens represent the same species and the same evolutionary significant unit (ESU).

While the inbreeding coefficient was significantly larger than zero on both islands, results suggest that, before the population crash, the Dominican population was larger than that of Montserrat, harbouring greater genetic variation. The island of Dominica is approximately eight times larger than Montserrat and both island biogeography theory and population genetics studies indicate that populations persisting on smaller and more-isolated islands tend to have lower genetic diversity than those living on larger and more-connected islands (e.g.^43^,^44^,^45^). It has been previously hypothesised that Amerindian dispersal from South America into the Lesser Antilles may explain the presence of mountain chickens on these islands^21^. If such hypotheses are correct, it could also be expected that Dominica would harbour more genetic variation as it is closer to South America than Montserrat. We did not test for this hypothesis explicitly and no mountain chicken samples from South America are known. Nevertheless, the genetic diversity of the Montserrat mountain chicken population is likely to have been further affected by volcanic events that periodically eliminated mountain chickens from pyroclastic sites on the island^46^,^47^. While prior to the chytridiomycosis-driven decline some differentiation between the mountain chicken populations in these islands was observed (F_ST_ ~ 0.2), it is likely that further population size reductions will exacerbate the differences between the relic populations, as well as further reducing the overall genetic variation of this species.

Within Dominica, a temporal trend in genetic diversity could be seen. Whilst comparisons of *pre-* and *post-* decline heterozygosity and inbreeding coefficients were not significant, the difference between *pre*- and *post-* decline allelic richness was close to significant (a metric found to be much more sensitive for detecting demographic change over short time scales^48^,^49^). Recently, genetic bottlenecks were detected in Bd-affected populations of the common midwife toad (*Alytes obstetricans*) in central Spain^15^. They were not, however, able to compare pre- and post- decline genetic diversity at the same location. In the present study we were unable to detect evidence of a genetic bottleneck in the *post-decline* population on Dominica using the program Bottleneck, despite seeing both a trend of loss of heterozygosity and marked differences in genetic diversity metrics for the *pre-* and *post-* decline populations. However, very recent bottlenecks often go undetected in genetic data using limited sample sizes, even when it is known that those bottlenecks did occur^50^. In our case, the *post-decline* dataset for the Dominican mountain chicken population was confined to 17 samples taken in 2014 and this sample size might not have been high enough to detect a genetic bottleneck signature.

On Montserrat, a comparison between the founders of the captive population (*Founders*) and the wild individuals sampled pre-decline (*Wild*) showed very little difference in expected heterozygosity or allelic richness, and genetic differentiation (F_ST_) between the two populations was zero. Also, the resampling analysis showed that the genetic diversity captured by the founder stock adequately represented the wild population, while the F_IS_ indicated no significant evidence of inbreeding. Captive breeding programs for wildlife conservation require careful genetic management to avoid further reductions of genetic diversity^51^. In this context, molecular analysis can be helpful in establishing the comparative diversity of wild and captive populations and in managing that genetic diversity in future captive generations^52^. In the case of the mountain chicken, care should be taken to ensure that the captive population continues to represent the founding gene-pool in subsequent generations (e.g. by controlling mating to avoid increasing inbreeding, and avoiding population decreases of the capture population), especially as eventual reintroduction is the primary aim^53^.

Shortly after the onset of the chytridiomycosis epidemic on each island, mountain chickens were provided full legal protection and their hunting was banned. The Dominican government also banned the import of amphibians or amphibian products following the discovery of chytridiomycosis on that island. During the seven-year gap between the emergence of chytridiomycosis in the Lesser Antilles (i.e. on Dominica) and its incursion to Montserrat, amphibian populations on the latter island were shown to be free from Bd infection and a risk analysis identified the most likely routes of introduction. Unfortunately, no identified mitigation measures were implemented. Capacity for sanitary measures that remove amphibian stowaways and contaminated soil from produce prior to export was developed in Dominica in 2007 to enable exports to the Lesser Antillean islands of Guadeloupe and Martinique. Had this requirement been imposed for produce shipped to Montserrat, the risk of Bd incursion would likely have been much reduced.

There is evidence of enzootic Bd infection in Caribbean tree frogs such as *Eleutherodactylus coqui* from Puerto Rico^54^ and *E. Johnstonei* on Montserrat (authors’ unpublished data) indicating some tolerance to Bd infection. The intentional or accidental transport of amphibians such as these between islands is a likely route of transmission of Bd throughout the Caribbean (http://www.mountainchicken.org/wp-content/uploads/2010/11/Chytridiomycosis-Management-Plan.pdf). The World Organisation for Animal Health (OIE) listed Bd in May 2008, making the pathogen internationally notifiable and thus subject to OIE standards to assure the sanitary safety of international trade in live amphibians and their products. Despite this, few if any of the OIE’s 180 member states have imposed relevant sanitary requirements on traded amphibians. This might be because, although it is a huge industry involving tens of millions of animals annually^55^, the international amphibian trade is generally unregulated and unrecorded^56^. Neither Dominica nor Montserrat are members of the OIE, although other island states with high levels of amphibian endemicity which would be threatened by Bd incursion are (e.g. Sri Lanka). If lessons to help the world meet its Aichi 2020 targets are to be learned from the demise of the mountain chicken, these member states should be implementing the necessary sanitary regulations and instigating Bd surveillance and control measures.

## Methods

### Study sites

Montserrat and Dominica are small volcanic islands in the Lesser Antilles chain in the Caribbean. Montserrat, approximately 102 km^2^, has a central active volcano creating its highest peak at 915 m. The Centre Hills to the north, with a maximum altitude of 740 m, are characterised by deep ‘ghauts’ (river valleys) in a radial pattern from a central peak^47^. The climate on Montserrat is characterised by two distinct seasons, a dryer, cooler season running from January to June and a wetter, warmer season from July to December. Dominica is larger, approximately 754 km^2^, and more mountainous than Montserrat, with steep interior slopes, most of which are covered in native forest. It has a tropical climate with year-round rainfall. Dominica lies approximately 150 km South East of Montserrat with the island of Guadeloupe situated between them.

### Population Monitoring

A full description of transect monitoring techniques is provided in ref. 19. Briefly, the relative mountain chicken population abundance was measured across multiple 10-metre-wide 200–250 m long transects on each island using VES. Two or more observers walked slowly along the transect midline shining torches into the vegetation on each side and every mountain chicken seen was recorded in a single dataset. Each survey was conducted shortly after nightfall when the frogs are active^19^. Each VES lasted approximately 1 hr per 200 m. At the beginning and end of each survey, relative humidity (%) and ambient temperature (^o^C) were recorded.

On Dominica, nine transects were established in 2002 in areas with mountain chicken populations, typically in lowland coastal areas. These were surveyed on an approximately monthly basis from August 2002 to March 2004. Surveys were conducted on eight occasions from January 2006 to July 2007. From January to September 2014, the established transects and an additional 13 (selected on local knowledge of historical distributions and patches of potentially suitable habitat) were surveyed on three occasions to search for any surviving mountain chickens. On Montserrat, mountain chickens inhabit upland areas in which 17 transects of 200 m were established in 1998; 13 were monitored regularly from 1999 to 2005: transects were surveyed four times in 1999, biannually (wet and dry seasons) from 2000 to 2005 and once per year in 2007, 2011 and 2012. From 2003, some transects were extended to a maximum of 400 m to capture a greater proportion of the available habitat at each site, with effort per transect-metre maintained.

Following the onset of declines on Montserrat, an extended 800 m length of the transect with the last known intact population (Fairy Walk) was surveyed using VES on a thrice-weekly basis from August 2009 to January 2010 as part of an amphibian chytridiomycosis treatment trial^42^. Only individuals in the treatment trial control group were included in the GLM of the Fairy Walk decline to omit any treatment effects. Ad-hoc monitoring of these transects was carried out between 2012 and 2014 to identify any remnant mountain chicken populations or individuals.

### Disease diagnosis

Systematic pathological examinations were conducted on frogs found dead during the initial stage of epidemic mortality on each island by examining formalin-fixed samples of hind toe and skin from the ventral pelvic area (drink patch) from each carcase. Following the emergence of chytridiomycosis on each island, the epidemic was tracked via skin-swabbing of frogs found sick or dead using rayon-tipped swabs^42^. Swabs were refrigerated until Bd analyses were undertaken in the laboratory. Fieldwork biosecurity measures were undertaken as recommended by the Amphibian and Reptile Groups, UK ARG Advice Note 4 (http://www.arguk.org/advice-and-guidance/view-category) to minimise Bd contamination of samples or the spread of pathogens between study sites by researchers. DNA was extracted from fresh/frozen hind toe and ventral pelvic skin samples and examined for the presence of Bd DNA using a Bd-specific qPCR^57^ but adding bovine serum albumin to the PCR mix to minimise any reaction inhibition^58^. Negative controls and four standards (100, 10, 1 and 0.1 zoospore equivalents) were included on each plate and all samples, standards and controls, were tested in duplicate. Samples were defined as positive if both duplicates showed a successful PCR product. However, if only one duplicate worked a third qPCR was run. If the third repeat showed no PCR product, the sample was considered negative. Genomic equivalent (GE) values for each sample were calculated by multiplying the mean quantity of the duplicate dilutions by 120 (4 μl from 60 μl total elute used to make 1 in 10 dilution (x15) and 5 μl of 40 μl dilution was used in qPCR (x8) [15 × 8 = 120]).

### Analysis of population data

To ensure encounter rates were comparable across transects of different lengths and between islands, a nightly average encounter rate per 200 m of transect was calculated, excluding two Dominican transects where no visual encounters were made. Generalised linear mixed models in R^59^ were used to assess temporal trends on Dominica between 2002–2004 with transect as random effect, assuming the counts were Poisson distributed, and using a logit link function. Transect surveys on Dominica in 2006 and 2007 were not included in the analysis as no individuals were seen during this period, however the model from the 2002–2004 data was used to predict the expected encounter rates in those years. On Montserrat, visual encounter trends from 1999 to 2005 also were analysed using a Poisson GLMM with a logit link and with transect as a random effect. To examine the trends at the intensively-monitored Fairy Walk, the pre-decline VES data for this site were modelled independently with a Poisson GLM with a logit link function, and the decline between August 2009 and January 2010 was modelled using a Poisson GLM with a logit link function.

The environmental variables collected for each survey, the number of observers, the season (Wet/Dry), and the average rainfall in the month of, and in the month prior to, the survey were included as covariates in the models to account for mountain chicken detectability. Models were developed using stepwise elimination of the least significant variable and a likelihood ratio test used to assess whether the model fit was reduced by exclusion of the variable compared to the model containing that variable at an alpha value of 0.05. This was continued until only variables that significantly improved model fit were retained.

### Range change estimates

As widespread systematic monitoring of the mountain chicken distribution was not available outside the transects, two standardised range metrics used in the red listing process^60^ were calculated: i) EOO and ii) AOO for both the pre-epidemic distribution and the present day distribution (definitions provided in Supplementary data 2). Each metric was independently calculated for each island.

We used Fig. 1 in ref. 61, which estimates the mountain chicken distribution prior to 2003 using polygons and points, to estimate its pre-epidemic distribution on Dominica. Any grid cell which was more than half filled with a polygon in the map in ref. 61 or occupied by a transect point was considered occupied. Present day estimates of the distribution on Dominica were generated using the data from the 2014 extended transects and personal communications with forestry staff. On Montserrat, pre-epidemic distributions were generated using transect data from the routine monitoring carried out from 1998–2007. Present day estimates on Montserrat were generated using the non-systematic monitoring carried out between 2012 and 2014 and personal communications with forestry staff. In both cases, grid squares of current distribution were randomly assigned to within a 3 square linear distance of their location to protect the location of the populations.

### Population genetics

**Figure 6:**
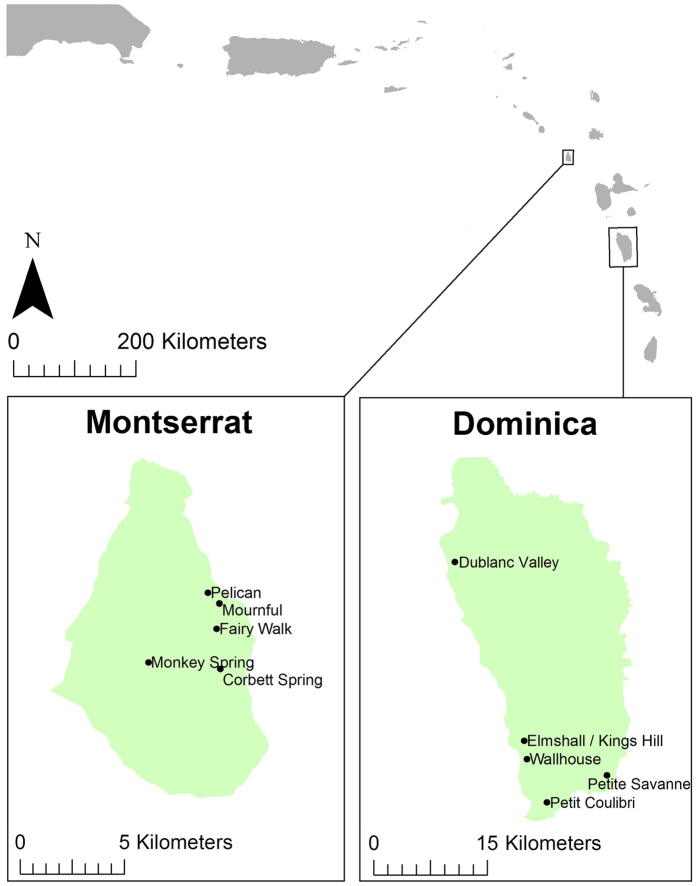
Location of pre-decline mountain chicken samples collected and used in genetic analysis. Figure created in Esri ArcMap 10.2. under license (www.esri.com).

To assess if there were differences in genetic variation between islands or between populations *pre-* and *post-* declines (including between wild and captive populations), genetic analyses were conducted using microsatellite markers. Blood, buccal swabs or tissue samples from live and dead wild mountain chickens were collected from Montserrat and Dominica (Fig. 6) between 2002 and 2014. For founders and first generation captive-bred individuals from Montserrat, buccal mucosa was sampled by swabbing with rayon-tipped swabs^62^ and stored in 99% ethanol. All extractions were performed using the Roche High Pure PCR Template Preparation Kit (Ref: 11796828001; Version 16). DNA from buccal swabs was extracted following the same tissue protocol with modifications as follows: swabs were removed and allowed to air dry for 5 minutes for ethanol to evaporate, after which they were placed in individual micro-centrifuge tubes with 40 μl Proteinase K and 200 μl tissue lysis buffer and incubated at 56 °C overnight. After this extended incubation, the tissue protocol steps were followed. Eluted DNA was stored at −20 °C.

Eight newly-developed polymorphic microsatellite markers were genotyped for each animal (Supplementary data 3 and Supplementary Table 3) and the resultant genotypes were checked for null alleles and allelic drop out using MicroChecker v.2.2.3^63^. Hardy Weinberg equilibrium and linkage disequilibrium, were calculated with Genepop v.4^64^,^65^, and observed and expected heterozygosity, unbiased allelic richness, and genetic differentiation among populations (F_ST_) were estimated with MSA v4.05^66^. Per locus and per population inbreeding coefficient (F_IS_) was estimated by FSTAT v.2.9.4.^67^.

For the *pre-* and *post-* decline genetic comparison of the Dominican samples we use a decline end-date of 2006 by which time no animals could be detected in the wild. The *post-decline* (recovering) population was defined as animals caught in 2014 when mountain chickens began to be detected on three of the historical transects in the absence of chytridiomycosis driven mortality. In the 2014 surveys, a large number of ~2 year old animals were detected alongside a small number of older animals, suggesting that breeding had occurred at increased levels in 2012, although no samples were collected in 2012 or 2013. Although 6 juveniles were found at one location in 2011, they were infected with Bd and disappeared shortly after (presumed to have died of chytridiomycosis) and so these animals were not considered part of a recovering population (authors’ unpublished data). As these animals were caught so long after the initial decline but before the apparent population recovery began, the animals were excluded from the *pre-*/*post- decline* comparison but included in the general interisland genetic diversity statistics. The *pre-*/*post- decline* groups of samples were compared using standard population genetic diversity statistics described above to assess if genetic diversity in the *post-decline* population was significantly smaller as a consequence of the Bd*-*induced population crash. Additionally, signatures of demographic contraction in the 17 wild Dominican individuals caught in 2014 were tested using the Wilcoxon test in the software Bottleneck v. 1.2.02^68^. For this analysis, 1000 simulations were carried out under the stepwise mutation model (S.M.M.,^69^) and the Two-Phased mutation model (TPM^70^) using default parameters.

### Representatives of the founder population

A resampling analysis was carried out to establish whether the Fairy Walk (Montserrat) individuals collected in 2009 used to establish the captive founder population (*Founders*), were representative of the genetic diversity in the larger wild population (*Wild*). For this purpose, we compared the average number of alleles per locus, observed and expected heterozygosity of the Fairy Walk individuals against 100,000 random samples of eleven individuals from the allele frequency distribution of the 74 wild individuals from Montserrat. We selected these statistics as they are affected by dramatic changes in effective population size^49^. In order to test for a difference, we examined whether the statistics for the observed data were within the 95 percentiles of the random sample distribution.

## Additional Information

**How to cite this article**: Hudson, M. A. *et al*. Dynamics and genetics of a disease-driven species decline to near extinction: lessons for conservation. *Sci. Rep.* **6**, 30772; doi: 10.1038/srep30772 (2016).

## Supplementary Material

Supplementary Information

## Figures and Tables

**Table 1:** Statistical descriptors of genetic variation.

**Population**	**n**	**ANA**	**AR**	**HO**	**HE**	**FIS**
Dominica	52	6.0	6.0	0.45	0.59	0.233*
Montserrat	120	4.8	4.3	0.49	0.59	0.167*
Dominica: *pre-decline*	29	5.9	5.2	0.49	0.61	0.197*
Dominica: *post-decline*	17	3.5	3.5	0.35	0.49	0.283*
Montserrat: *Founders*	11	3.5	3.5	0.51	0.58	0.116
Montserrat: *Wild*	74	4.4	3.3	0.48	0.59	0.180*

^*^Indicates F_IS_ values significantly different from 0 (P < 0.001). Populations deviating from Hardy Weinberg Equilibrium are indicated with bold F_IS_ values. Among the Dominican samples, six collected between 2011 and 2012 were excluded from the *pre-* and *post-* population decline analyses as they did not clearly belong to either group. Among the Montserrat individuals, 35 samples correspond to animals that had been captive-bred and thus were excluded from the comparison between the *Founders* and *Wild* samples.

Descriptor values are averaged across all 8 loci. For each population the corresponding sample size is shown (n), the observed average number of alleles per locus (ANA), allelic richness (AR), the observed heterozygosity (H_O_), the expected heterozygosity (H_E_), and the population’s inbreeding coefficient (F_IS_).
