# Corrigendum: Dynamics and genetics of a disease-driven species decline to near extinction: lessons for conservation

**DOI:** 10.1038/srep40110

**Published:** 2017-01-11

**Authors:** M. A. Hudson, R. P. Young, J. D’Urban Jackson, P. Orozco-terWengel, L. Martin, A. James, M. Sulton, G. Garcia, R. A. Griffiths, R. Thomas, C. Magin, M. W. Bruford, A. A. Cunningham

Scientific Reports 6: Article number: 30772; 10.1038/srep30772 published online: 08 03 2016 updated: 01 11 2017.

In this Article, R. A. Griffiths is incorrectly listed as being affiliated with ‘Durrell Wildlife Conservation Trust, Les Augres Manor, Trinity, Jersey, Channel Islands, UK’ The correct affiliation is listed below:

Durrell Institute of Conservation and Ecology, School of Anthropology and Conservation, University of Kent, Canterbury, Kent CT2 7NR, UK.

