# Supplementary material for: Dynamics and genetics of a disease-driven species decline to near extinction: lessons for conservation
Source: Sci Rep. 2016 Aug 3;6:30772. doi: 10.1038/srep30772 (PMC4971493; doi:10.1038/srep30772)
Supplement: Supplementary Information [file srep30772-s1.docx]

**Supplementary information**

**Dynamics and genetics of a disease-driven species decline to near extinction: lessons for conservation.**

Hudson, M.A., Young, R.P., D’Urban Jackson, J., Orozco-terWengel, P., Martin, L., James, A., Sulton, M., Garcia, G., Griffiths, R.A., Thomas, R., Magin, C., Bruford, M.W., and Cunningham A.A.

**Supplementary Table 1. Mountain chickens found dead or with severe signs of chytridiomycosis on Dominica between 2003-2004**

| **Date** | **Site** | **Number dead** | **Number with signs of chytridiomycosis** |
| --- | --- | --- | --- |
| 13/12/2002 | Gallion | 2 | 0 |
| 13/01/2003 | Bagatelle | 0 | 1 |
| 24/01/2003 | Galion | 0 | 2 |
| 30/01/2003 | Elmshall | 1 | 0 |
| 01/02/2003 | La Haut | 1 | 0 |
| 17/02/2003 | Bois Cotelette | 1 | 0 |
| 19/02/2003 | Petit Coulibri | 1 | 1 |
| 25/02/2003 | Petit Coulibri | 0 | 1 |
| 25/02/2003 | Soufriere | 1 | 1 |
| 06/03/2003 | Dublanc Valley | 1 | 0 |
| 19/03/2003 | Bagatelle | 0 | 1 |
| 26/03/2003 | Bois Cotelette | 0 | 1 |
| 28/03/2003 | Dublanc Valley | 3 | 1 |
| 01/04/2003 | La Haut | 4 | 0 |
| 26/12/2003 | Milton Estate | 1 | 0 |
| 17/01/2004 | Elmshall | 1 | 0 |
| 12/02/2004 | Upper Kings Hill | 1 | 0 |
| 14/04/2004 | Coulibistre | 1 | 0 |
| 15/06/2003 | Fond St. Jean | 2 | 4 |
| 26/06/2003 | Fond St. Jean | 1 | 5 |

**Supplementary Table 2. Summary statistics of all loci in all populations**

NA = Number of alleles, H_O_ = Observed heterozygosity, H_E_ = Expected heterozygosity. Loci significantly (p=<0.05) deviating from HWE indicated in shaded cell, red = heterozygote deficiency, green = heterozygote excess. Significant (p= <0.05) F_IS_ values are shown in bold.

|  | **Locus** | **NA** | **H_O_** | **H_E_** | **F_IS_** |
| --- | --- | --- | --- | --- | --- |
| **Founders** | 0673_p1 | 3 | 0.545 | 0.450 | -0.224 |
|  | 759A_p1 | 6 | 0.818 | 0.688 | -0.200 |
|  | 0867_p1 | 3 | 0.545 | 0.437 | -0.263 |
|  | 2969_p1 | 3 | 0.727 | 0.662 | -0.103 |
|  | 3035_p1 | 2 | 0.364 | 0.485 | 0.259 |
|  | 1628_p1 | 3 | 0.000 | 0.606 | **1.000** |
|  | 3956_p1 | 3 | 0.182 | 0.589 | **0.701** |
|  | 7957_p1 | 5 | 0.909 | 0.684 | -0.351 |
|  |  |  |  |  |  |
| **Wild** | 0673_p1 | 4 | 0.473 | 0.441 | -0.072 |
|  | 759A_p1 | 6 | 0.838 | 0.724 | **-0.158** |
|  | 0867_p1 | 6 | 0.581 | 0.528 | -0.101 |
|  | 2969_p1 | 3 | 0.622 | 0.650 | 0.044 |
|  | 3035_p1 | 3 | 0.500 | 0.529 | 0.055 |
|  | 1628_p1 | 3 | 0.041 | 0.588 | **0.931** |
|  | 3956_p1 | 4 | 0.081 | 0.516 | **0.844** |
|  | 7957_p1 | 6 | 0.740 | 0.741 | 0.001 |
|  |  |  |  |  |  |
| **Post Dom** | 0673_p1 | 3 | 0.529 | 0.558 | 0.053 |
|  | 759A_p1 | 6 | 0.294 | 0.627 | **0.539** |
|  | 0867_p1 | 5 | 0.412 | 0.592 | 0.311 |
|  | 2969_p1 | 3 | 0.353 | 0.426 | 0.176 |
|  | 3035_p1 | 3 | 0.235 | 0.314 | 0.256 |
|  | 1628_p1 | 2 | 0.353 | 0.471 | 0.256 |
|  | 3956_p1 | 2 | 0.000 | 0.148 | **1.000** |
|  | 7957_p1 | 4 | 0.647 | 0.770 | 0.164 |
|  |  |  |  |  |  |
| **Mont** | 0673_p1 | 4 | 0.550 | 0.514 | -0.071 |
|  | 759A_p1 | 8 | 0.825 | 0.745 | **-0.108** |
|  | 0867_p1 | 6 | 0.533 | 0.501 | -0.064 |
|  | 2969_p1 | 3 | 0.622 | 0.656 | 0.052 |
|  | 3035_p1 | 4 | 0.492 | 0.506 | 0.029 |
|  | 1628_p1 | 3 | 0.025 | 0.571 | **0.956** |
|  | 3956_p1 | 4 | 0.100 | 0.477 | **0.791** |
|  | 7957_p1 | 6 | 0.765 | 0.722 | -0.059 |
|  |  |  |  |  |  |
| **Pre Dom** | 0673_p1 | 7 | 0.621 | 0.743 | 0.168 |
|  | 759A_p1 | 8 | 0.759 | 0.736 | -0.031 |
|  | 0867_p1 | 10 | 0.759 | 0.814 | 0.069 |
|  | 2969_p1 | 5 | 0.370 | 0.387 | 0.044 |
|  | 3035_p1 | 4 | 0.357 | 0.611 | **0.420** |
|  | 1628_p1 | 4 | 0.138 | 0.356 | **0.616** |
|  | 3956_p1 | 5 | 0.148 | 0.546 | **0.733** |
|  | 7957_p1 | 4 | 0.793 | 0.702 | -0.132 |
| **Dom** | 0673_p1 | 7 | 0.596154 | 0.677931 | 0.122 |
|  | 759A_p1 | 8 | 0.615385 | 0.710232 | 0.135 |
|  | 0867_p1 | 10 | 0.634615 | 0.744586 | **0.149** |
|  | 2969_p1 | 5 | 0.36 | 0.391919 | 0.082 |
|  | 3035_p1 | 5 | 0.313725 | 0.558144 | **0.44** |
|  | 1628_p1 | 4 | 0.230769 | 0.434653 | **0.472** |
|  | 3956_p1 | 5 | 0.088889 | 0.453433 | **0.806** |
|  | 7957_p1 | 4 | 0.769231 | 0.720127 | -0.069 |

**Supplementary data 1. Mitochondrial DNA sequences**

A 463bp fragment of the cytochrome oxidase subunit I gene was amplified for 32 individuals (16 randomly chosen samples from each island) using universal amphibian primers developed by Che *et al.* (2012): Chmf4, 5’ - TYT CWA CWA AYC AYA AAG AYA TCG G – 3’;Chmr4, 5’ - ACY TCR GGR TGR CCR AAR AAT CA - 3’. To produce a final PCR reaction volume of 10µl, the following reagents were included, 5µl Qiagen Multiplex mix, 0.6µl of 0.1pmol/µl reverse and forward primer, 1µl of DNA and 2.8µl of nuclease free ddH_2_O. The PCR conditions were as follows: 95⁰C for 15min, followed by 35 cycles of: 94⁰C for 1min, 56⁰C for 1min, extension for 1min at 72⁰C; and a final extension of 10min at 72⁰C. Un-purified PCR products were sequenced in both directions using the Eurofins MWG Operon's DNA sequencing service. Sequences were verified using Sequencher ® (version 5.1) and subsequently aligned by the ClustalW multiple sequence alignment algorithm (Thompson *et al.,* 2002) implemented in BioEdit Sequence Alignment Editor (Hall, 1999; version 7.1.11) with the default parameters. A single haplotype was present in all individuals (GenBank accession number KX579492).

Che, J., H.-M. Chen, J.-X. Yang, J.-Q. Jin, K. Jiang, Z.-Y. Yuan, R. W. Murphy, and Y.-P. Zhang. 2012. Universal COI primers for DNA barcoding amphibians. *Molecular Ecology Resources* 12:247–58.

Hall, T.A., 1999. BioEdit: a user-friendly biological sequence alignment editor and analysis program for Windows 95/98/NT. *Nucleic acids symposium series* 41: 95-98).

Sequencher® version 5.4.5 sequence analysis software, Gene Codes Corporation, Ann Arbor, MI USA [http://www.genecodes.com](https://webmail.zsl.org/owa/redir.aspx?SURL=I-2rrrE9IEmlWyV7eBLqPfF88vwomj0sCWOl5vUWblpWz8PodbDTCGgAdAB0AHAAOgAvAC8AdwB3AHcALgBnAGUAbgBlAGMAbwBkAGUAcwAuAGMAbwBtAA..&URL=http%3a%2f%2fwww.genecodes.com)

Thompson, J.D., Gibson, T. and Higgins, D.G., 2002. Multiple sequence alignment using ClustalW and ClustalX*. Current protocols in bioinformatics,* pp.2-3.

**Supplementary data 2. Definitions of extent of occurrence (EOO) and area of occupancy (AOO)**

The EOO is defined as “The area contained within the shortest continuous imaginary boundary which can be drawn to encompass all the known, inferred or projected sites of present occurrence of a taxon” (IUCN, 2001). This was calculated by drawing a minimum convex polygon around the midpoints of all transects on which mountain chickens had been observed. This likely represented an overestimate as it almost certainly included areas of unsuitable habitat. The AOO is defined as “The area within its 'extent of occurrence', which is occupied by a taxon, excluding cases of vagrancy” (IUCN, 2001). This was calculated by overlaying a 500 m grid on both islands and assuming full occupancy of a grid square if mountain chicken presence had been confirmed on a transect which had its midpoint within a grid square. A 500 m grid size was chosen as it represents the best current guess of the home range size of the mountain chicken (authors’ unpublished data).

Reference:

IUCN (International Union for the Conservation of Nature), 2001. IUCN Red List Categories and Criteria version 3.1.

**Supplementary data 3. Microsatellite primer development**

Microsatellite primers for the mountain chicken were isolated commercially by Ecogenics (Switzerland). Eight polymorphic markers (Supplementary Table 3) were then standardised using a Qiagen Multiplex PCR Kit using the following reaction mix: 5 µl of Multiplex mix, 0.2ul of 0.2 pmol/µl of forward primer, 0.2µl of 0.2pmol/µl of reverse primer, 1µl of DNA and 3.6µl of nuclease free ddH_2_O. PCR conditions were as follows: initial denaturation at 95⁰C for 15min, followed by 35 cycles of: 30s at 94⁰C, 90s at specific annealing temperature (Supplementary Table 3), extension for 60s at 72⁰C; and a final extension of 30min at 72⁰C. All genotyping was performed at DNA Sequencing and Services (Dundee) and microsatellite scoring was performed using GeneMapper (v4).

The microsatellite genotypes will be submitted to Dryad upon acceptance of this manuscript for publication, and the corresponding accession code will be provided here.

**Supplementary Table 3. Polymorphic microsatellites identified for *Leptodactylus fallax***

For each marker the annealing temperature (Tm ⁰C), number of observed alleles (NOA), repeat motif (mot), PCR products allele size range in base pairs (range), combinations of primers for multiplexing (M) are shown.

| **Primer Code** | **Tm (°C)** | **Primer sequence (5’-3’)** | **NOA** | **Mot** | **Range** | **M** |
| --- | --- | --- | --- | --- | --- | --- |
| Lepfal_010673 | 62 | AGCAATTCTTGTTGCCTCCC | 5 | (TAGA) | 217-241 | 3 |
|  |  | AGCCTAAGTTCTTGCAGGGC |  |  |  |  |
| Lepfal_015759A | 64 | AAGATCAGCCAGGGACAGAC | 10 | (TTTC) | 189-234 | 3 |
|  |  | CACTGTGATATTTAGGGGTGC |  |  |  |  |
| Lepfal_000867 | 64 | CGTGAGAAAGACTAGGGCAC | 10 | (TAGA) | 200-244 | 1 |
|  |  | AAAAGGGAGCACTCCACAGG |  |  |  |  |
| Lepfal_002969 | 60 | AGCATCACAGGGAACCAGTC | 5 | (AC) | 169-191 | 2 |
|  |  | GCTCCTGAAGTACAAACGCC |  |  |  |  |
| Lepfal_003035 | 62 | ACATACAGAACTGCTTACATGTCC | 5 | (TG) | 126-134 | 1 |
|  |  | GCTTTGTCACTGGCTCCAAG |  |  |  |  |
| Lepfal_011628 | 60 | ATGATTGGCCCCAGTGTATG | 4 | (CA) | 207-234 | 2 |
|  |  | GATCGCAGAACCTGGACCTC |  |  |  |  |
| Lepfal_013956 | 58 | AGCGTTCGATTAGTAGCTGTG | 5 | (AC) | 134-162 | 2 |
|  |  | AGTTCACCCCAACGTAGGAC |  |  |  |  |
| Lepfal_017957 | 58 | TGTATGATGTGGGCCTTCCC | 6 | (TG) | 189-242 | 1 |
|  |  | CACCACTGAAATAACCTATCATTTGTC |  |  |  |  |
